# Context matters: using reinforcement learning to develop human-readable, state-dependent outbreak response policies

**DOI:** 10.1098/rstb.2018.0277

**Published:** 2019-05-20

**Authors:** W. J. M. Probert, S. Lakkur, C. J. Fonnesbeck, K. Shea, M. C. Runge, M. J. Tildesley, M. J. Ferrari

**Affiliations:** 1 Big Data Institute, Li Ka Shing Centre for Health Information and Discovery, Nuffield Department of Medicine, University of Oxford, Oxford OX3 7LF, UK; 2 Department of Biostatistics, Vanderbilt University, Nashville, TN 37203, USA; 3 Department of Biology, Center for Infectious Disease Dynamics, The Pennsylvania State University, University Park, PA 16802, USA; 4 US Geological Survey, Patuxent Wildlife Research Center, Laurel, MD 20708, USA; 5 Department of Life Sciences and Mathematics Institute, University of Warwick, Coventry CV4 7AL, UK

**Keywords:** machine learning, reinforcement learning, outbreak response, vaccination, optimal control, FMD

## Abstract

The number of all possible epidemics of a given infectious disease that could occur on a given landscape is large for systems of real-world complexity. Furthermore, there is no guarantee that the control actions that are optimal, on average, over all possible epidemics are also best for each possible epidemic. Reinforcement learning (RL) and Monte Carlo control have been used to develop machine-readable context-dependent solutions for complex problems with many possible realizations ranging from video-games to the game of Go. RL could be a valuable tool to generate context-dependent policies for outbreak response, though translating the resulting policies into simple rules that can be read and interpreted by human decision-makers remains a challenge. Here we illustrate the application of RL to the development of context-dependent outbreak response policies to minimize outbreaks of foot-and-mouth disease. We show that control based on the resulting context-dependent policies, which adapt interventions to the specific outbreak, result in smaller outbreaks than static policies. We further illustrate two approaches for translating the complex machine-readable policies into simple heuristics that can be evaluated by human decision-makers.

This article is part of the theme issue ‘Modelling infectious disease outbreaks in humans, animals and plants: epidemic forecasting and control’. This theme issue is linked with the earlier issue ‘Modelling infectious disease outbreaks in humans, animals and plants: approaches and important themes’.

## 1. Introduction

Computational models of disease spread and control have been widely used in preparedness planning for outbreaks of infectious disease to both forecast outbreak severity [1–4] and decide among competing control interventions [5–10]. A conventional approach for determining optimal interventions has been to evaluate the expected performance of different potential interventions across a large number of stochastic simulations [5,11–13]; thus, interventions are ranked according to expected performance over all possible outbreaks. In a real outbreak, however, only one realization is of concern and there is no guarantee that the control action that performs best, on average, is also optimal for any specific outbreak. The current state of an outbreak dramatically reduces the set of all possible future trajectories. The decision-maker should therefore seek to find the optimal action conditional on the current state of the outbreak [14,15]. Thus, we define a ‘state-dependent policy’ as a rule set that defines an action to be taken, conditional on the current state of the dynamical system (here an infectious disease outbreak).

While it is intuitive that outbreak response should be dependent on the specific realization of an outbreak, generating optimal policies *a priori* is computationally challenging. A brute force solution to this problem requires enumerating all possible states of the epidemic system—e.g. for all spatial distributions of susceptible, infected and controlled premises at a given point in time—and for each state simulating all possible futures conditional on each action one could take. The results of such an exercise could then be stored in a look-up table that returned the action that performed best over all future simulations for any given state. For even relatively small problems, this approach is computationally intractable and grossly inefficient, at least in part because a large amount of computation would be expended evaluating states that are only rarely observed (though function approximation methods can help to overcome this; e.g. [16,17]).

In lieu of identifying optimal policies, other computational methods have been used to provide guidance for management. Regression methods, for example, have been used to model associations between predefined predictors and the occurrence of a disease outbreak [18,19]. Similarly, network-based approaches have been implemented in contact tracing efforts to retrospectively learn about outbreak propagation [20–22]. Formally, a decision-analytic framework with a well-defined objective to be optimized is required to ensure optimality. Methods such as dynamic programming offer an exactly optimal solution, and heuristics such as simulated annealing or genetic algorithms can provide a competitive (though not guaranteed optimal) solution [23–25].

**Figure 1.**
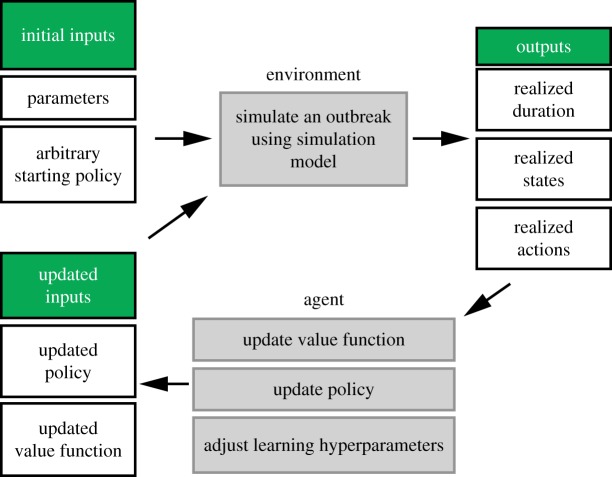
Schematic of Monte Carlo control for solving RL problem (adapted from Sutton and Barto [26]). (Online version in colour.)

Reinforcement learning (RL), a class of machine learning algorithms, and Monte Carlo (MC) control use feedback from real or simulated systems to estimate optimal actions for states that are likely to be visited, as governed by a dynamic model [26]. RL algorithms involve the interaction of some environment, which encapsulates a system that is to be managed, with an agent that learns how to manage the environment through direct experience (figure 1). The state can be defined as a statistic of the environment, such that it includes all the information that is relevant to model the decision-making problem, which in an outbreak setting might include attributes such as the number of infected individuals, the available store of vaccine, etc. The agent chooses actions to take in order to achieve its objective, while responses to the agent's actions occur in the environment, including rewards. The output from an RL control algorithm is a policy, providing a rule set that maps states of the system to actions to take when the system is in that state [26]. Underpinning a policy is a value function, *Q*(*s,a*), which returns the expected total future value of following an estimated optimal policy, across the space of all likely future states, conditional on selecting action *a* from the current state *s*. RL methods iteratively improve the estimate of *Q*(*s*,*a*) by repeated simulation using feedback from the system, choosing actions that balance the precise estimation of the current best policy with the search for better policies. For instance, actions may be chosen using an epsilon-greedy algorithm whereby the current best action is chosen most of the time but a small (epsilon) proportion of the time, an exploratory random action is chosen to diversify and potentially enhance understanding of the outcomes [26]. A stochastic simulation model ensures that likely future states are explored, and the RL algorithm ensures that the range of actions is evaluated for each state. This approach is especially advantageous in larger decision spaces, where it may be infeasible to visit each state–action pair many times. Computational runtime can be improved by using function approximation in place of a look-up table, allowing for generalization across neighbouring states and actions, and the corresponding reward. Thus, without sampling the full state-action space, RL may reveal novel patterns learned over the course of training [27].

Although we are not aware of a formal proof of MC control algorithms guaranteeing an optimal policy [26,28], MC control and RL have an impressive track record, having been successfully applied to win games, such as backgammon [16], Atari computer games [27] and Go [29], and in the control of robots [30]. In these applications, the intended user of the policy is a computer, rather than a human, and so, because of the ease of storing and looking-up large multi-dimensional policies on a computer, there is little or no constraint on the number of state variables used to construct the policy. These areas of application have thus been concerned with demonstrating how RL methods can be used to replace human decision-makers. Here, our aim is to generate policies that will support human decision-making by presenting a low-dimensional representation of a context-dependent policy that can help to inform more nuanced decision-making in outbreak situations.

We present two case studies that use RL and MC control to develop state-dependent response policies in the context of a livestock outbreak, based on the dynamics of the 2001 foot-and-mouth disease (FMD) outbreak in the UK. In the first case study, we consider the initial stages of an outbreak, where the state space is relatively small, and generate state-dependent RL policies using deep Q-learning. In the second case study, we consider a spatially large-scale epidemic and illustrate the development of a state-dependent policy using a reduced two-dimensional summary of the full state space; thus, the resulting policy is readable as a two-dimensional mapping of summary states to control interventions. Finally, we discuss the challenges and opportunities for the application of RL to outbreak control policies.

## 2. Material and methods

### (a) The foot-and-mouth disease system

FMD, a viral disease of livestock, is a pathogen for which epidemiological models have been widely applied. For FMD-free countries, such as the UK and USA, emergency preventative measures aim to avoid the large economic ramifications of such outbreaks that result from the cessation of trade [31,32]. We use a stochastic, individual-based model of FMD spread, where the modelling unit is a premises (farm), based on Keeling *et al*. [5] and Tildesley *et al*. [33] (details are presented in the electronic supplementary material). The probability of virus spread between farms is modelled as both an increasing function of farm size and a decreasing function of physical/geographical separation [8]. Each premises can be in one of four epidemiological states: susceptible, exposed, infectious or removed/immune. A fixed period of time is assumed from virus exposure to being infectious, from virus exposure to notification of FMD infection (in case study 2), and between notification and the time at which culling on a premises begins [5,33].

### (b) Case study 1: deep Q-networks

**Figure 2.**
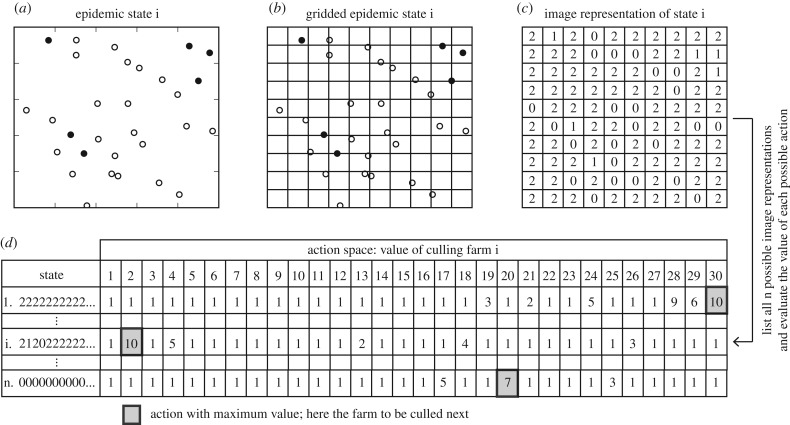
Three preprocessing steps to construct the state (*a*–*c*). (*a*) We first plot the farms and identify farm-level infection statuses: black = infected, white = susceptible; then (*b*) overlay a grid to ‘pixelate’ the landscape so that no more than one farm occupies a pixel; then (*c*) construct a two-dimensional array of farm-level infection status: 0 = no farm, 1 = infected farm, 2 = susceptible farm. (*d*) Schematic of utility table, with flattened states as rows and actions as columns. Shaded cell represents the action with the highest utility for the state in each row. The RL methods in both case studies seek to approximate the value function represented in this ‘look-up table’ representation of the state-action space.

We used deep Q-networks (DQN) to derive FMD outbreak response policies on three different landscapes. DQN combine RL with convolutional neural networks (CNN), which serve as function approximators. DQN are particularly suited to image data inputs owing to their use of CNN, which are able to extract low-level feature information and combine it with other features to represent abstract concepts [28,34], such as nonlinear value functions. In this case study, the objective was to terminate the outbreak as quickly as possible, with minimal costs, specified by the immediate reward *r* in the action-value function. We defined the state at time *t* using an image of the disease outbreak to capture the spatial relationships between farm locations (figure 2).

To better illustrate the motivation for DQN, we highlight scenario 1, in which the landscape comprised 30 farms, ranging in size from 25 to 500 animals (figure 2). We assumed that only one farm could be culled per day, thus 30 possible culling actions were available at the first time step (figure 2). Under this state–action space, expected utility updates for each state–action pair would be computationally intensive, both in terms of computational time and memory storage. Specifically, with three possible states for each farm, there are 3^30^ possible states and 30 possible culling actions at the first time step. A look-up table would therefore require 6.17 × 10^15^ cells to be updated (e.g. rows in figure 2*d*). DQN were used instead to approximate the action-value function and are well suited to the image input. The FMD simulation model was coded in Python 3.6 and the DQN were implemented using TensorFlow and the Keras submodule (see electronic supplementary material for pseudo-code and details).

The action–value function *Q*(*s,a*) allows us to evaluate how a particular action, in the context of the state of the environment, contributes to achieving the encoded objective. The immediate reward (*r*) is a key component in calculating the expected utility in DQN:

$$Q(s,a)=E_{s^{\prime }}[r+\gamma \mathrm{ma}x_{a^{\prime }}Q(s^{\prime },a,)|s\;,a],$$

where the prime superscript indicates the subsequent state, *s*, (after taking action *a*). In our case study, *r* was defined as:

$$\begin{array}{rll} r & = & -100(\mathrm{number}\;\mathrm{of}\;\mathrm{farms}\;\mathrm{culled})\;+\\ & & -500(\mathrm{number}\;\mathrm{of}\;\mathrm{times}\;\mathrm{out}\;\mathrm{break}\;\mathrm{resurges}\;\mathrm{due}\;\mathrm{to}\\ & & \;\mathrm{neglecting}\;\mathrm{the}\;\mathrm{culling}\;\mathrm{of}\;\mathrm{exposed}\;\mathrm{farms})\\ & & +\;\mathrm{number}\;\mathrm{of}\;\mathrm{remaining}\;\mathrm{cattle}\;\mathrm{by}\;\mathrm{the}\;\mathrm{end}\;\mathrm{of}\;\mathrm{management}. \end{array}$$

Resurgence here refers to an outbreak going from 0 to 1 infected farm due to the transition of an undetected exposed farm. Here, the weights were manually chosen to penalize culling of farms more severely than individual cattle and put a severe penalty on stopping culling when farms that are exposed, but not yet detected, are still present.

### (c) Case study 2: applying reinforcement learning to a summary state space

In the second case study, our objective is to minimize the duration of an FMD outbreak over a landscape of 4001 randomly distributed farms of 500 cattle each. Parameters governing both the seeding of the outbreak and of control interventions were similar to previous modelling studies (e.g. [8,35,36]). Each simulation used the same four seeding premises.

We assume that managers can implement one of two control interventions—ring culling or ring vaccination—after an initial period of silent spread and obligatory culling of infected premises (IPs). Ring culling or ring vaccination designate premises to be culled (or vaccinated) that are within a predefined radius (here 3 km) of premises that are confirmed to be infected with FMD. The rate of ring culling was limited to 200 animals culled per day per premises. Disposal of culled carcasses was assumed to occur at a rate of 200 animals per day per premises. We assumed there was a global constraint on the number of animals culled per day because of the public health and public perception concerns of a large build-up of undisposed carcasses [37]. This assumption limits new culling when the number of carcasses reaches a ‘carcass constraint’. For vaccination, we assumed that immunity was conferred 7 days after vaccination date and that vaccine efficacy was 100%. Premises exposed to FMD before conferral of immunity were assumed to progress to an infectious state. Vaccination was delivered at a rate of 200 animals per day for each premises and it was assumed there was a global constraint on the number of vaccines administered per day of 10 000 doses.

Here we assume that a single decision must be made to implement ring culling or ring vaccination at 3 km after 12 days of silent spread and 7 days of IPs culling. Thus, while the decision space is much smaller (two actions) than in case study 1, the number of premises (4001 farms) is much larger. We therefore generate a state-dependent policy that is human readable by constraining the state space to a two-dimensional summary. Two summary state variables were used to construct the policies: (1) number of IPs at the decision time point, and (2) area of the outbreak at the decision time point. Area of the outbreak was represented by the convex hull that included any culled, confirmed infected or exposed premises. We chose the number of IPs as a state variable because (a) it is correlated with time until the first detection, which has been cited elsewhere as important for predicting the severity of outbreaks [38]; (b) it changes throughout the course of an outbreak; and (c) ring culling and ring vaccination strategies take place in areas surrounding IPs so the application and outcome of these actions will vary according to the number of infected premises. The area of the outbreak was chosen as a state variable because it increases monotonically as an outbreak progresses and it is therefore possible, in combination with the number of IPs, to distinguish between the start of an outbreak and the end of an outbreak using these two states.

We used epsilon-soft MC control to construct control policies using 100 000 outbreak simulations [26]. Epsilon, which determines how often to choose a currently non-optimal action, was set at 0.1 (see electronic supplementary material for alternative values of epsilon). Pseudo-code for this algorithm is provided in the supplementary material. RL algorithms and epidemiological simulation models were coded in Python 3.5.2 using the packages numpy (1.14.1), pandas (0.22.0) and Cython (0.28.3). The RL code for case study 2 is available at the following repository: https://github.com/p-robot/context_matters.

## 3. Results

### (a) Case study 1

**Figure 3.**
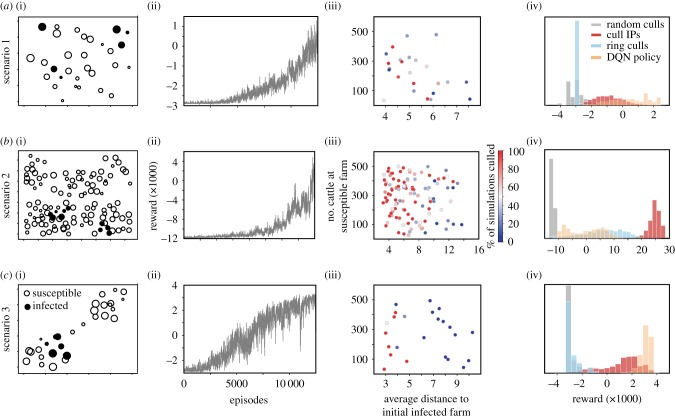
The spatial distribution of farms for the three scenarios (rows *a*–*c* respectively); circle size scales with farm size (column i). (*a*(ii)-*c*(ii)) Performance of DQN, in terms of the reward, r, for each case study during training. (*a*(iii)-*c*(iii)) The frequency at which susceptible farms were culled during 2000 simulations of testing (colours) plotted as a function of the mean distance to the initially infected farms and the farm size. (*a*(iv)-*c*(iv)) The distribution of rewards for 2000 simulations using either the best DQN policy, a policy of culling farms at random (e.g. a null policy), or a policy of culling infected premises (IPs) or a policy of ring culling.

We illustrate DQN using three scenarios, each reflecting a different population spatial structure. The first was a small-scale example with 30 farms distributed uniformly at random on a 10 × 10 km grid with six farms initially infected (figure 3*a*(i)). We trained the DQN for 10 000 episodes, and the associated total reward trajectory illustrates a gradual increase in total reward over the course of training (figure 3*a*(ii)). This suggests that the DQN was able to choose better farm culling sequences following 10 000 episodes of training. Though the final optimal policy is difficult to illustrate due to the size of the state–action space, we can summarize the behaviour of the optimal policy by illustrating how often each farm was culled under many simulations of management under the optimal policy (figure 3*a*(iii)). The farms that were culled tended to be both larger and closer to the initially infected farms than the average, which is consistent with the underlying transmission model. The reward trajectory does not indicate convergence to an optimal policy, though over only 10 000 episodes the policy performs, on average, better than both a policy of either culling farms at random (which may remove as yet undetected premises) or a policy of culling only known IPs (which necessarily implements culling only after farms can potentially transmit, figure 3*a*(iv)), or a policy of ring culling. Due to the one-farm-per-day culling constraint, ring culling was implemented by ranking farms to be culled by the closest distance to an infected premises [8].

The second scenario was a larger simulation with 120 farms distributed uniformly at random on a 15 × 15 km grid with 10 farms initially infected (figure 3*b*(i)). Here we assume that all of the cattle at any five farms per day could be culled. As above, the DQN failed to converge on an approximately optimal policy (figure 3*b*(ii)), though, as in case study 1, the best policy found consistently prioritized culling of farms near infected premises that were larger than average (figure 3*b*(iii)). The best DQN policy did not perform better than a policy of culling only IPs (figure 3*b*(iv)) or ring culling. The possible state–action space for this case study is much larger, 2.16 × 10^59^ possible state–action pairs, and would likely require a considerably longer training period.

The third scenario also used 30 farms, with 6 farms initially infected and a daily culling capacity of one farm, but assumes a clustered spatial distribution of farms with the initial infections all in a single cluster, the farms in the lower left corner (figure 3*c*(i)) and a strongly distance-dependent transmission kernel; thus, infection must pass through the ‘bridge’ farms to reach the second patch. Here, the reward for the DQN policy appears to plateau over a 10 000 episode training period (figure 3*c*(ii)). Here the optimal policy is simpler than above and the DQN learned to cull the bridge farms. In 2000 simulations of testing, at least one of the two bridging farms was culled 1744 times while it was still susceptible. Both bridging farms were always culled (100% of simulations) before they became infected, i.e. culled while susceptible or exposed, preventing the outbreak from spreading to the second cluster of farms. The pattern of susceptible farms culled was more correlated with location than farm size (figure 3*c*(iii)). The resulting DQN policy here performs better than both random culls, culling of IPs and ring culling because of the benefit of culling bridging premises while they are still susceptible (figure 3*c*(iv)). Additional challenges with DQN are discussed in the electronic supplementary material, figure S2.

### (b) Case study 2

**Figure 4.**
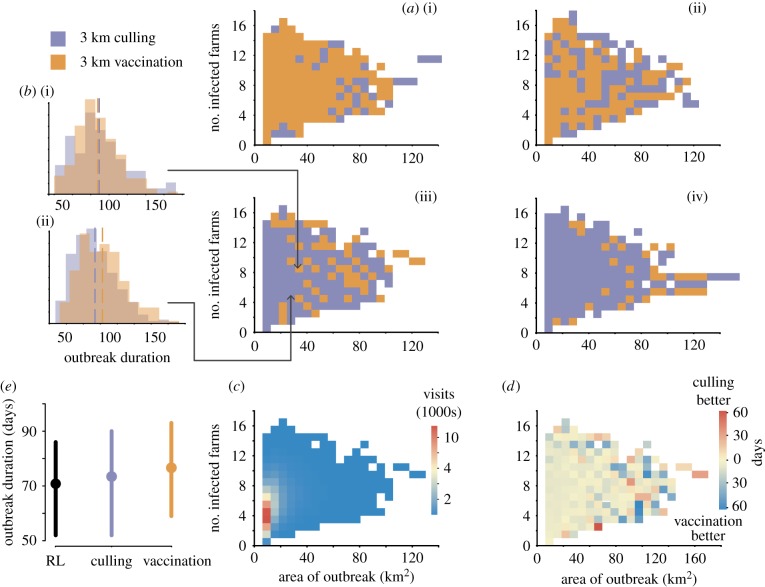
Optimal policies to minimize outbreak duration as a function of outbreak area and number of infected premises. (*a*) Output policy for minimizing outbreak duration for different carcass constraints: (i) 11 000, (ii) 13 000, (iii) 15 000, (iv) 17 000 carcasses. (*b*) Histogram of outbreak duration following enacting ring culling or ring vaccination for states highlighted in *a*(iii). (*c*) Heatmap of the frequency of visits to each state throughout all simulations used to construct the RL policy in *a*(iii). (*d*) Heatmap of the difference in outbreak duration when using ring culling at 3 km or ring vaccination at 3 km for each state for the carcass constraint illustrated in *a*(iii). (*e*) Distribution of outbreak duration for simulations (using culling constraints ranging from 10 000 to 20 000; see electronic supplementary material, figure S6 for all policies) managed using the RL policy compared with static policies of ring culling and ring vaccination; circles give mean, bars give IQR. (Online version in colour.)

Because we summarized the state space for this case study in two dimensions, using the spatial extent of the outbreak and the number of farms infected, we can plot a map of the resulting RL policy (figure 4*a*), indicating the best action to take for each position in the summary state space. For stringent carcass constraints (less than or equal to 12 000), vaccination was the optimal control intervention for almost all states (figure 4*a*(i)). With ample resources (carcass constraint greater than or equal to 18 000), culling was almost always optimal regardless of the state of the outbreak (figure 4*a*(iv); see electronic supplementary material for additional carcass constraints). Between these extremes in resources, the optimal policy was composed of a mix of culling and vaccination depending upon the number of infected premises and area of the outbreak at the time point in question (figure 4*a*(ii),(iii),*b*,*d*; see electronic supplementary material, figure S6 for policies for all carcass constraints from 10 000 to 20 000). Over much of the summarized state space, the expected difference in actions was small, but the difference was large for extreme, though rare, outbreaks (figure 4*c,d*). Notably, for intermediate culling constraints, culling is preferred for outbreaks that are small, in a number of premises, relative to their areal extent (i.e. more densely clustered), but vaccination is more likely favoured when outbreaks are sparse (figure 4*a*(iii),(iv); electronic supplementary material, figure S6). On average, over all carcass constraints (electronic supplementary material, figure S6), the RL strategy resulted in shorter outbreaks (figure 4*e*; mean difference (IQR): 2.6 (0,4) days); note that the small average difference occurs because outbreaks, where switching of actions is recommended and may achieve large differences in outcome, are relatively rare (figure 4*d*). Developing general rules using RL remains an active area of research (e.g. [39]). The RL policies presented here are conditional on the starting conditions used for these simulations. In the electronic supplementary material, we show that these policies perform on par with, but not better than, the static strategies when outbreaks are seeded with random starting conditions that the learner was not exposed to.

## 4. Discussion

Historically, management interventions have indeed changed as outbreaks have progressed [31]. Constructing optimal policies that anticipate these changes, however, is a non-trivial computational task. Here we have shown that RL may be a useful tool for developing state-dependent policies that outperform static strategies and yet nevertheless can easily be interpreted by human decision-makers. The RL approach is a significant improvement over the conventional comparison of static policies, as it allows the discovery of optimal state-dependent control policies [26] and the generation of non-intuitive control policies that are not limited to consideration only of control policies that can be defined *a priori*.

Through our first case study, we illustrated that RL can efficiently estimate approximately optimal state-dependent policies for outbreak response control problems where an exhaustive search through the state space is computationally infeasible. Though the resulting policy is itself too complicated to illustrate simply, we showed that the state-dependent RL policy produces intuitive control for simple landscapes—prioritizing culling on farms that are at high risk of infection because of proximity, or high potential for onward spread because of large farm size. On the clustered landscape (scenario 3), the RL policy also identified the ‘bridge nodes’ between clusters as optimal sites for culling to prevent spread between clusters, highlighting the dependence of the optimal policy on the landscape [40]. Thus, *post hoc* analysis of RL policies can help to develop heuristics that can inform policy decisions.

In our second case study, we presented an approach that uses MC control to generate low-dimensional, human-readable state-dependent policies. We used simulation first to illustrate the epidemic settings (here a constraint on culling capacity) for which a state-dependent policy results in an expected benefit, and second to show that *a priori* definition of a simple summary state representation can help to guarantee a human-readable policy. A human-readable policy is not as easily obtained with DQN (figure 2*d*). Utilities can be assigned to each state–action pair, thought of as a look-up table; however, visualization of this table becomes difficult when the state is large.

There are three key limitations in delivering practical state-dependent policies: computational challenges, challenges in interpretation and communication of the output policies, and challenges in implementation.

RL itself offers a solution to the computational challenge of an exhaustive search through the state–action space; here we illustrated the development of state-dependent policies using simulations over only a small fraction of the state space. However, as seen in our first case study, long training periods may be necessary to achieve approximately optimal policies for large state spaces; two weeks of training was required for scenario 2 of case study 1. Deploying such methods in a real outbreak may require parallelization of simulation models or highly efficient computational code, and the RL algorithms themselves may require tuning of hyperparameters (see further discussion in the electronic supplementary material). Some of these details could be tested in non-outbreak settings to improve reaction to real outbreaks.

The interpretation of policies, particularly those that are generated in settings with a high-dimensional state space, and the communication of output policies to policymakers remains a challenge. There are two pathways to producing human-readable policies: (1) by generating a full policy, then using statistical methods to reduce the policy itself to a manageable dimension (as in case study 1); and (2) by simplifying the state space prior to searching for the optimal policy (as in case study 2). We note that there is no guarantee that a summary state space (e.g. case study 2) will necessarily result in an improved expected performance benefit or a tractable state-dependent policy. Thus, the choice of this summary state representation requires careful thought and expert input. An RL policy can itself be subjected to further analysis; machine learning methods, such as classification and regression trees, have been used to highlight variables that have a large influence on the severity of outbreaks (e.g. [38]) and to provide a starting point for the systematic selection of state variables.

The translation of the theoretical gains from using state-dependent control into real-world action requires operational mechanisms that may not yet exist; e.g. pre-existing data sharing agreements and transfer to allow real-time state updating or logistical infrastructure for switching response teams between control activities. Modelling and optimization can be used in scenario-planning exercises before any outbreak to investigate state-dependent preparedness plans and communicate findings to policymakers. During emergencies, systems must already be in place to allow rapid communication and dissemination of data on the state of the outbreak, and resources must be available to enable redeployment, or repurposing, of personnel.

Several research questions are opened up by our approach. It remains to be determined what is the limiting complexity of a policy; for example, what is the best low-dimensional representation of the state space, or what is the upper limit of complexity of the state space, to ensure the resultant policy is both interpretable and logistically feasible in the field? Simple state-dependent policies already exist for emergency response in the form of flow diagrams (e.g. [41] figure E p. 72, or [42]) and previous research regarding likelihood of adoption of computer-based aids for clinical decision-making identified the ability of a system to justify the advice it was providing as most important [43]. The likelihood of adoption of state-dependent policies may depend critically on the complexity and communication of the policy, and recent interest in ‘explainable AI’ may be the catalyst for initiating such investigations [39].

Here, we have assumed the model is known, but in a real outbreak, parameter estimation and/or model selection may occur simultaneously with the construction of RL policies. It may be possible, however, to use state variables representing a measure of model uncertainty, thereby allowing RL methods to identify control actions that would reduce uncertainty through time (e.g. using active adaptive management; [44,45]).

Here, we have ignored the additional operational costs of measuring the state space (e.g. surveillance) and of switching among management actions (e.g. overhead costs or costs such as travelling between premises). Additional work to account for these costs is critical to the full evaluation of these methods. Finally, the choice of null strategies against which to assess the performance of state-dependent policies is not always easy. In case study 2, the comparison was against precedents in the modelling literature, but for our case study 1, the choice of null strategy does not have a precedent, and some potential baseline strategies, such as no management, are unrealistic comparisons in an outbreak scenario given there are minimum legal intervention requirements under EU law [46].

RL, coupled with epidemiological models, presents an exciting new avenue to develop optimal control policies. Rather than replacing human decision-makers, we propose applications that augment human decision-making by either using a computer-readable policy to develop practical policy heuristics or directly generating a human-readable policy. Thus, RL has the potential to provide well-supported yet tractable state-dependent policy summaries to facilitate decision-making in times of crisis.

## Supplementary Material

Additional Methods

## Data accessibility

The datasets and code for case study 2 (https://github.com/p-robot/context_matters) supporting this article have been uploaded as part of the electronic supplementary material.
