# Supplementary material for: Context matters: using reinforcement learning to develop human-readable, state-dependent outbreak response policies
Source: Philos Trans R Soc Lond B Biol Sci. 2019 May 20;374(1776):20180277. doi: 10.1098/rstb.2018.0277 (PMC6558555; doi:10.1098/rstb.2018.0277)
Supplement: Additional Methods [file rstb20180277supp1.docx]

Supplementary information for **Context Matters: using reinforcement learning to develop human-readable, state-dependent outbreak response policies**

[Additional Methods: Case study 1 1](#_Toc534656816)

[Deep Q-Network learning algorithm 1](#_Toc534656817)

[DQN hyperparameters 2](#_Toc534656818)

[FMD Model equations and parameters 5](#_Toc534656819)

[Parallelization 6](#_Toc534656820)

[Software Implementation 6](#_Toc534656821)

[Additional Methods: Case study 2 8](#_Toc534656822)

[FMD Transmission model 8](#_Toc534656823)

[Control interventions 8](#_Toc534656824)

[Reinforcement learning algorithm 9](#_Toc534656825)

[Convergence 12](#_Toc534656826)

[Sensitivity to Starting Conditions 12](#_Toc534656827)

[References: 14](#_Toc534656828)

# Additional Methods: Case study 1

## Deep Q-Network learning algorithm

The goal of deep Q-networks (DQN) is the same as that of RL: construct an optimal policy for a given Markov decision process (MDP). DQN combines Q-learning, an RL method to update the action value function based on experiences, with convolutional neural networks (CNN) to estimate and store network weights. The algorithm begins with a simplified version of the Bellman optimality equation:

$$Q^{*}(s,a) = E_{s'}[r + \gamma max_{a'}Q^{*}(s',a')| s,a]$$

By incorporating convolutional neural networks into the Q-learning algorithm, the optimal action-value function is approximated, $Q^{*}(s,a) \approx Q(s,a; \theta)$where $\theta$ represents the collection of convolutional neural network weights. As with traditional Q-learning, the estimate of $Q^{*}(s,a)$ improves with increased agent experience. The improvement in the estimate mainly occurs through improvement of the convolutional neural network weights, $\theta$. With each iteration of training, the squared error loss is calculated:

$$L(\theta) = E_{s,a,r}(E_{s'}(r + \gamma max_{a'}Q^{*}(s',a';\theta^{-})) - Q(s,a,;\theta)){}^{2}$$

where $\theta^{-}$refers to a previous set of weights. Following the convolutional neural network (CNN) algorithm, partial derivatives of the squared error loss are calculated with respect to $\theta$, and stochastic gradient descent can be performed to update the network weights.

There are additional components to the DQN algorithm, in comparison to traditional Q-learning: experience replay and target updating. With experience replay, an agent can experience the effects of taking “realistic” actions from given states without having to execute those actions in real time [1]. These actions are realistic because they were previously taken from the same given state. Experience replay saves the experience $(s_{t},a_{t},r_{t},s_{t+1})$ from each time step in a double ended queue, or “deque”, *E* of pre-specified length, *N*. Each time the convolutional neural network is fit, a random sample of experiences from *E* is drawn, and is used in the weight updating process. The deque *E* contains only the *N* most recent experiences to ensure the estimates are using the most relevant data. Consecutive experiences are highly correlated, thus randomising previous experiences reduces correlation in the estimates of $Q(s,a; \theta)$[2].

Target updating refers to using a separate network for the target, $Q(s',a'; \theta^{-})$. The target network and the Q-network, for $Q(s,a; \theta)$, initially have the same weights, $\theta$. The Q-network weights are updated with each time step, while the target network weights remain the same. After *C* time steps, the Q-network is cloned and equated to the target network for the next *C* time steps. This method may require more episodes of training, but can greatly improve the stability of training and prevent optimal policy divergence [3].

FIGURE S1: Deep Q-Network algorithm adapted from [2]

1. Initialise experience replay memory *E* to capacity *N*
2. Initialise action value function *Q* with random weights 𝜃
3. Initialise target action-value function $\hat{Q}$with random weights 𝜃^-^ = 𝜃
4. For each episode:
   1. Initialise and pre-process *s*
   2. Until *s* is terminal
      1. Select action *a_t_* randomly with probability ℇ, otherwise select *a_t_* using ${a_{t} = {argmax}_{a} Q(s,a; \theta)}$
      2. Implement *a_t_* and observe *r_t_*
      3. If the daily culling capacity has not been reached
         1. *s_t+1_* determined by the new farm culled and state pre-processing
      4. If the daily culling capacity has been reached
         1. Evolve outbreak and pre-process to observe *s_t+1_*
      5. Store (*s_t_, a_t_, r_t_, s_t+1_*) in *E*
      6. Sample random mini batch of (*s_t_, a_t_, r_t_, s_t+1_*) in *E*
      7. Set $y_{i}=r_{i}$if episode terminates at *i + 1*
         1. Otherwise $y_{i}=r_{i}+\gamma max_{a_{t+1}}\hat{Q}(s_{t}, a_{t+1}; \theta)$
      8. Perform stochastic gradient descent on $(y_{t} - Q(s_{t}, a_{t}; \theta)){}^{2}$w.r.t network parameters, 𝜃
      9. Every *C* steps let $\hat{Q}= Q$

## DQN hyperparameters

Parameter selection in deep reinforcement learning methods is still understudied. Mnih et al. [2] mentioned that, in their study, the hyperparameters were selected by performing an informal search, and a systematic grid search would result in high computational costs. There were many hyperparameters to tune in the first case study, and this excludes searching for a proper functional form of continuous hyperparameters (Tables S1, S2). This investigation used an informal hand-tuning method for the first case study, and similar hyperparameters as in Mnih et al. [2] for the remaining case studies. Manual parameter tuning is highly subjective. It is almost guaranteed that if two individuals were given the same initial data, the final tuned models would be very different due to the large number and functional forms of hyperparameters. The alternative to manual tuning and grid search methods are formal optimisation methods. There have been some advances in optimising neural network hyperparameters, such as using genetic algorithms or expected improvement in Gaussian Processes [4,5]. However, these approaches have not been implemented in a DQN environment. The actual value of the hyperparameter may also affect the trajectory of learning (FIGURE S2)

Table S1: Values of RL-based hyperparameters used in the first case study

|  | **Definition** | **Scenario 1** | **Scenario 2** | **Scenario 3** |
| --- | --- | --- | --- | --- |
| **Initial epsilon** | Initial value of $\epsilon$in $\epsilon$-greedy algorithm | 1 | 1 | 1 |
| **Final epsilon** | Final value of $\epsilon$in $\epsilon$-greedy algorithm | 0.01 | 0.01 | 0.01 |
| **Epsilon decay function** | Function describing how $\epsilon$decays with each time step | Linear | Linear | Linear |
| **Experience replay start size** | #times policy is run before experience replay begins | 100 | 100 | 100 |
| **Experience replay memory size** | #samples stored with each experience replay | 2000 | 2000 | 2500 |
| **Target network update frequency** | #times target network is matched with current network | After every episode | After every episode | After every episode |
| **Discount rate** | Degree of importance of long/short-term rewards | 0.99 | 0.99 | 0.99 |
| **#Episodes for training (stopping criteria)** | Criteria to stop training | 10000 | 10000 | 8000 |

Table S2: Values of CNN-based hyperparameters in first case study

|  | **Definition** | **Scenario 1** | **Scenario 2** | **Scenario 3** |
| --- | --- | --- | --- | --- |
| **#Hidden layers** | #time nonlinear transformations occur on input | 4 | 4 | 4 |
| **#Nodes in dense layers** | #variables in linear combination | 155 | 155 | 155 |
| **#Filters in convolutional layers** | #features to be extracted | 32, 64, 64 | 32, 64, 64 | 32, 64,64 |
| **Patch size** | Dimensions of window in convolutional layer | 3x3 | 3x3 | 3x3 |
| **Activation function** | Function (linear/nonlinear) to transform previous layer of data | ReLu, linear | ReLu, linear | ReLu, linear |
| **Mini batch size** | #samples for model fit | 32 | 32 | 32 |
| **Learning rate** | Rate of abandoning old beliefs | 0.0001 | 0.0001 | 0.0001 |
| **Optimiser** | Process to search parameter space | RMSprop | RMSprop | RMSprop |
| **Epochs** | #times sample is forward and backwards propagated | 1 | 1 | 1 |


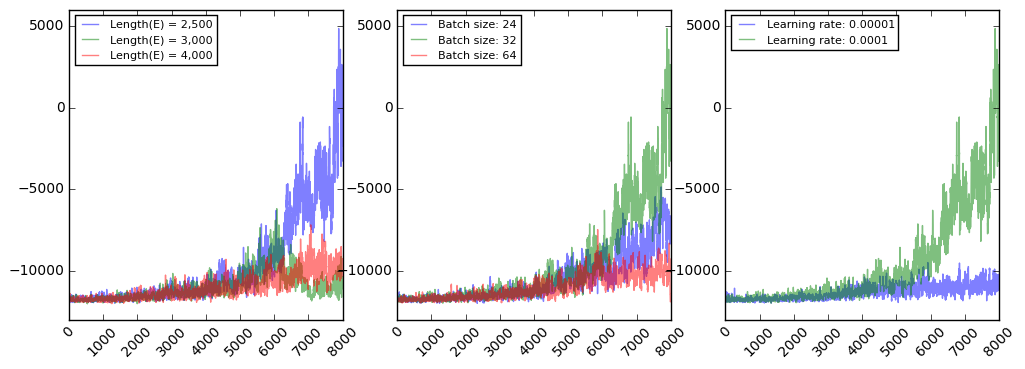


FIGURE S2: Trajectories of total reward during training for third scenario in first case study. One hyperparameter is changed in each panel, with all other hyperparameters remaining the same. The trajectories of total reward can be very different depending on hyperparameter values.

## FMD Model equations and parameters

Foot-and-mouth disease (FMD) outbreaks were simulated using an individual-based FMD over a 10 by 10 km landscape model as specified in the main text. The rate at which a susceptible farm became infected was represented by:

$\lambda_{t}= N_{t}s_{cattle}\sum_{j \in infectious} N_{j}{\tau_{cattle}K(d_{ij})}$ (1)

where *N* represents the number of cattle at a farm, *s* represents cattle susceptibility to the disease, $\tau$represents cattle transmissibility of the disease, *K* represents the transmission kernel, and $d_{ij}$represents the distance between the susceptible farm *i* and the infectious farm, *j*. Parameters used in each scenario are given in Table S1. The distance kernel for scenarios 1 and 2 decline gradually and is permissive of long-distance infections. The distance kernel used in scenario 3 declines more sharply with distance.

Table S3. Parameters for FMD transmission model

| **Outbreak Parameters** | **Scenario 1** | **Scenario 2** | **Scenario 3** |
| --- | --- | --- | --- |
| *s* | 8.4 x 10^-5^ | 8.4 x 10^-5^ | 4.9 x 10^-6^ |
| $\tau$ | 8.4 x 10^-5^ | 8.4 x 10^-5^ | 4.9 x 10^-6^ |
| $K(d_{ij})$ | $\frac{1}{distance + 400}$ | $\frac{1}{distance + 400}$ | $\frac{1}{\pi(distance + 1)}$ |

## Parallelization

An intuitive solution to address a larger decision space in DQN would be to train the agent for a larger number of episodes. DQN involves a Q-update, i.e. updating the expected reward based on: the immediate reward obtained from visiting the most recent state, and an estimate of the expected reward for the next state using the current model. Due to this architecture, DQN is serial in nature. Thus, serially training an agent for a larger number of episodes would present some challenges on run time. In the first case study, training the DQN in the third scenario took two weeks to complete in serial. More training would be required to achieve some stability in total reward (Figure S2). Even after training the DQN in the second scenario for the maximum amount of time in Advanced Computing Center for Research and Education (ACCRE) at Vanderbilt University, for two weeks, the immediate reward does not converge (Figure S3). DQN reached peak, but unstable performance, during earlier times in training. Chavez et al. [6] explored a parallelization alternative for DQN, involving Downpour stochastic gradient descent. This process involves an omniscient parameter server that stores a global copy of the DQN model. It also has “worker nodes" to perform updates on the DQN model. This parallelization scheme could be considered in future work for larger decision spaces, and would ideally help DQN surpass IP performance.

## Software Implementation

Outbreak state dynamics models were written in Python 3.6, using the NumPy and SciPy libraries. Deep Q-learning models were specified using the TensorFlow framework for deep learning [7], specifically the Keras submodule. Model fitting was performed on a high-performance computing cluster in the ACCRE. A repository containing the simulation and DQN code is available at: <https://github.com/fonnesbeck/dqn_rl_outbreak_response>.


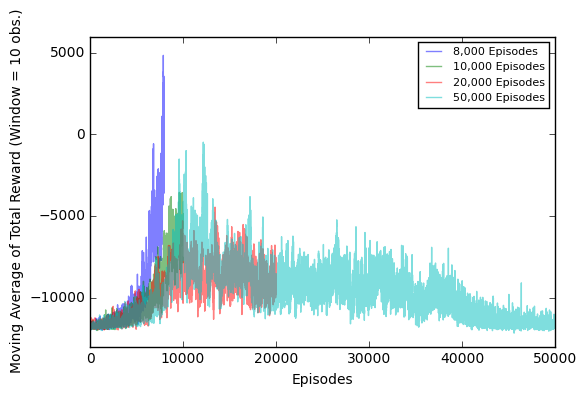


FIGURE S3: Total reward trajectory for second scenario, uniform landscape with 120 farms. Four instances of training were implemented with all hyperparameters the same, the only change is the duration of training. The instance with 8,000 episodes of training was used for analysis. When the DQN was trained for 50,000 episodes (which took two weeks to run in serial) we see a decline in the total reward trajectory. Ideally, with an infinite amount of training we should see some stability in the total reward. However, there are also challenges in hyperparameter tuning. Optimal hyperparameter tuning may involve some form of grid search method, and there are many parameters (and functional forms of parameters) within CNN and RL that need to be tuned, and can affect performance.

# Additional Methods: Case study 2

## FMD Transmission model

The transmission model followed a previously published individual-based model of foot-and-mouth disease [8,9] similar to the first case study. The infectious pressure on a particular susceptible farm at time *t* is given by equation (1). Per-capita transmissibility of cattle, ${\tau_{cattle}}$, was set at 10.5 and per-capita susceptiblity of cattle, $s_{cattle}$, was set at 1. The distance kernel, K, was based on that presented in Diggle [10; equation 10]. The values used in this kernel were phi = 0.05121725, kappa = 0.43693777, nu = 0.03315413, and rho = 0.0.  This kernel was truncated to return a value of 0.006265 for distances smaller than 0.11, and beyond a distance of 60.0 it returned a value of zero. This kernel is included in the code presented in the repository: https://github.com/p-robot/context_matters. This complete analysis was performed with the empirical kernel from Keeling et al. (2001) and the results were qualitatively the same.  The empirical kernel was not presented here due to confidentiality of the data.

No within-herd spread was simulated and between-farm spread was modelling. Exposed premises had a latent period of 4 days, after which they became infectious, and it was assumed it took an additional 5 days until a farm was confirmed infected with FMD and control was implemented. IP culling took place on any confirmed infected premises. Simulated outbreaks continued until there were 1) no exposed or infectious premises, 2) no premises currently being vaccinated, and 3) no premises with non-disposed carcasses present. When all these conditions were met, the simulation was stopped and duration of the outbreak (in days) was recorded.

## Control interventions

Ring culling and ring vaccination took place around infected premises within a designated radius (figure S4).


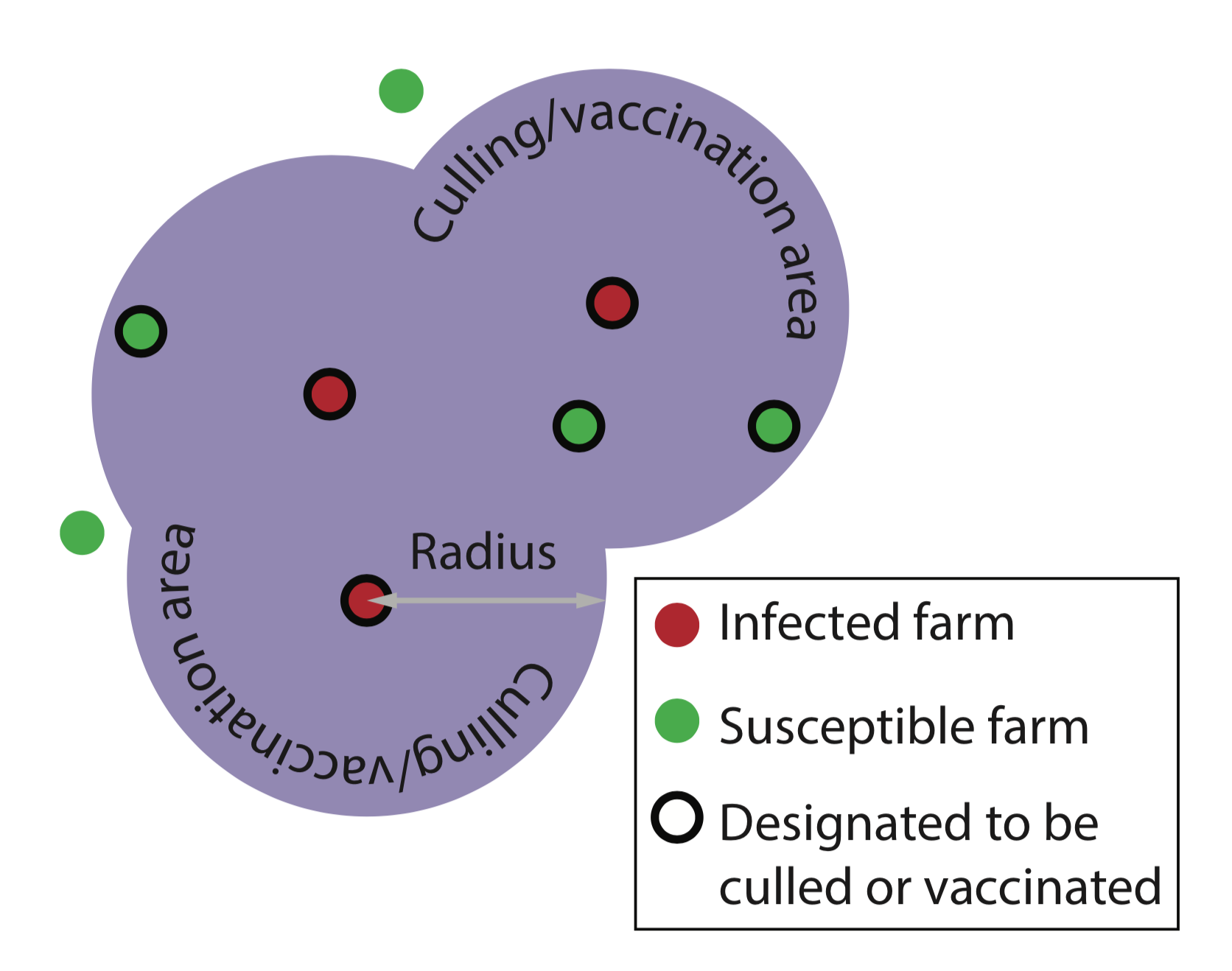


Figure S4: Schematic of ring culling/vaccination with culling/vaccination area shaded.

Culling was prioritised by first determining which premises are susceptible or exposed (but not confirmed infected) and within the culling radius around IPs, and then prioritising culling (up to the carcass constraint) based upon 1) IP or not (IPs are prioritised before ring culls), 2) whether the premises are currently being controlled or not (i.e. active culling prioritised before those in the queue), and 3) reporting date (earlier reporting date takes priority), 4) distance to the IP that earmarked them to be culled (further away is prioritised; i.e. “outside in” from [10]). Vaccination was prioritised in a similar fashion to culling (disregarding criterion 1 above). Premises that become infected while waiting to be vaccinated are assumed to become exposed and follow a standard route to becoming infectious and being detected. Any premises being vaccinated which are discovered to be infected have vaccination activities ceased and become IPs.

## Reinforcement learning algorithm

Figure S5 defines the epsilon-soft on-policy Monte Carlo control algorithm used in case study 2. The two state variables used were 1) the number of infected premises and 2) the area of the outbreak (the convex hull of all premises either infected or under control). Actions were either cull at 3 km around infected premises or vaccinate at 3 km around infected premises. The reward used to update the algorithm was outbreak duration of the simulated FMD outbreaks using the above-described individual-based model. Epsilon was set at 0.1, following canonical examples of reinforcement learning in [11]. Policies presented in the main text were constructed using 100,000 outbreak simulations. Policies constructed using only 40,000 outbreak simulations or epsilon = 0.4 were broadly consistent (Figure S6). Training of the RL policies with 100,000 outbreak simulations took roughly 12 hours on a single node of a high-performance computing cluster.

We also developed context dependent policies for a smaller vaccine capacity (5000 doses per day) and carcass constraints ranging from 10000 to 20000 (Figure S6). Overall, lower vaccine capacity favoured culling over more of the state space and the proportion of the state space that favoured culling increased with increasing carcass constraint. Lastly, we considered a policy that allowed 3 possible actions: 3 km ring culling, 3 km vaccination, and IP culling only. The resulting policy was very similar to the two-action policy (Figure S6) and IP culling was only favoured for outbreak states that had few infected farms over a wide area. We note that these were rare states that were infrequently visited (e.g. Figure 4D) and may reflect poor convergence in this region.

Figure S5: Epsilon-soft on-policy Monte Carlo control [11] for control of an FMD outbreak.

1. Initialise Value(s,a) a value function for all state and action combinations.
2. Initialise an array Returns(s,a) as an array of outbreak durations for all state and action combinations.
3. Initialise an arbitrary policy, pi(s,a) = 1/(Number of actions), for all s, a.
4. Repeat:
   1. Simulate an outbreak of 12 days of silent spread and 7 days of IP culling
   2. Choose and action, a, based upon policy (pi) and current state, s
   3. Simulate until the end of the outbreak, record outbreak duration, d.
   4. Append outbreak duration d to Returns(s,a).
   5. Update value function Value(s,a) = average(Returns(s,a))
   6. Update pi:
      1. a* = argmax_a(Value(s,a))
      2. For all a
         1. pi(s,a) = 1 - epsilon + epsilon/(Number of actions) if a = a*
         2. pi(s,a) = epsilon/(Number of actions) if a ≠ a*

Figure S6. (Left) RL policy plots for alternative vaccination and carcass constraints. Purple indicates states where 3 km culling is the best action, orange indicates states where 3 km vaccination is the best action. Panels indicate different combinations of carcass constraints and vaccination capacity. (Right) Illustrates the impact of changing RL parameters (epsilon and the number of training simulations) on the state-dependent RL policy for the case with vaccine capacity of 10000. Panels top to bottom indicate increasing carcass constraints. Colours are as in left panels.

## Convergence

Convergence of the value function is dependent upon the number of visits to each state, as dictated by the dynamic model and the number of times each action is taken in each state (controlled by the epsilon parameter).  Those states that were visited often (such as some of the initial states) were estimated quickly.  This is illustrated in Table S4 for two states in the policy presented in figure 4A.1 (carcass constraint of 11,000);

Table S4. Estimated outbreak duration as a function of duration of training for two states. % runs visited indicates the percent of training runs that visited this state. Carcass constraint for this simulation was 11,000.

|  | % runs | Number of Simulated Outbreaks for Training (1000s) | | | | |
| --- | --- | --- | --- | --- | --- | --- |
|  | visited | Action | 40 | 100 | 200 | 300 |
| 8 infected premises,  6.2 units square | 3% | Culling | 73.3 | 74.0 | 73.7 | 73.5 |
|  |  | Vaccination | 70.3 | 71.2 | 70.8 | 70.8 |
| 11 infected premises,  24.8 units square | 0.3% | Culling | 81.3 | 82.4 | 75.5 | 75.8 |
|  |  | Vaccination | 72.9 | 74.8 | 75.9 | 76.6 |

We note that the overall pattern of state-dependent policies is roughly consistent for the runs presented in figure S6. The transition from vaccination favored policies, to mixed policies, to culling favored policies occurs at similar culling constraints for the 40,000 and 100,000 training runs and is independent of the tuning parameter epsilon. We note that the policy for culling constraint of 19,000 has clearly not converged with only 40,000 runs. The rightmost column of figure S6 includes IP culling as a possible action, an intuitively inferior action for minimising outbreak duration.  In this instance the RL algorithm did not choose IP culling as optimal in any states that were visited more than a couple of times; that is, in parts of the state space where it had a chance to reasonably investigate the performance of different actions.

Convergence would be quicker if function approximation was used to approximate the value function with a surface (instead of using a look-up table as was used here) and convergence can also be helped by training the RL algorithm in areas that are not seen often (i.e. deliberately starting the simulated outbreaks as large outbreaks when training the learner).

## Sensitivity to Starting Conditions

An important aspect of reinforcement learning algorithms are their ability to extrapolate beyond the scenarios they were trained upon.  The management performance of the RL policy was simulated on 1000 outbreaks using a single randomly chosen seed case as the starting condition (figure S7).  The seed cases were chosen within 10 units of the centre of the landscape to avoid issues with calculating the area state on the borders of the landscape.  All other simulation parameters were kept as described in the main text for each particular policy that was used (i.e. those trained on a carcass constraint of 11,000 were simulated in an environment with a carcass constraint of 11,000).  In these simulation experiments, due to the algorithm using a look-up table, the RL algorithm encountered many states that it had not encountered before.  In this case, the state closest (in Euclidean distance) to the observed state was used to look-up the value function and find the action to implement.  For the four policies illustrated in figure 4, the RL algorithm performed worse than, or on par with, any of the single static policies at reducing outbreak duration (Table S5).  The RL policies were never the worse performing policy.  Thus, this indicates that while there may be some generality to the patterns discovered by the RL algorithm – they are not so specific that they underperform both static strategies with alternate starting conditions – developing robust and generalizable context dependent policies remains a challenging area of future research. Using function approximation to represent the value function (as opposed to a look-up table) might provide better performance when extrapolating beyond the training set.

Table S5. Mean outbreak duration from 1000 simulated outbreaks managed using the RL and static policies for random starting conditions. The minimum duration for each culling constraint is highlighted in bold.

|  | Outbreak Duration | | |
| --- | --- | --- | --- |
| Culling Constraint | RL | Culling | Vaccination |
| 11,000 | 42.5 | 44.5 | **41.7** |
| 13,000 | **40.6** | 41.3 | **40.6** |
| 15,000 | 39.9 | **39.7** | 41.5 |
| 17,000 | 39.2 | **37.0** | 41.3 |

# References:

1. Lin, L.J. (1993), Reinforcement learning for robots using neural networks, PhD the- sis.

2. Mnih et al. (2015) Human-level control through deep reinforcement learning. Nature. 518. 529-533 (doi:10.1038/nature14236)

3. Lillicrap, T. P., Hunt, J. J., Pritzel, A., Heess, N., Erez, T., Tassa, Y., Silver, D. and Wierstra, D. (2016), Continuous control with deep reinforcement learning, *International Conference on Learning Representations* .URL**:** *https://arxiv.org/pdf/1509.02971.pdf*

4. Fridrich, M. (2017), Hyperparameter optimization of artificial neural network in cus- tomer churn prediction using genetic algorithm, *Trends of Economy and Management* .

5. Bergstra, J., Bardenet, R., Bengio, Y. and Kegl, B. (2011), Algorithms for hyperparameter optimization, *NIPS* .

6. Chavez, K., Ong, H. Y. and Hong, A. (2015), Distributed deep q-learning.

7. Abadi, Martín, Ashish Agarwal, Paul Barham, Eugene Brevdo, Zhifeng Chen, Craig Citro, Greg S. Corrado, et al. 2016. “TensorFlow: Large-Scale Machine Learning on Heterogeneous Distributed Systems.” *arXiv [cs.DC]*. arXiv. http://arxiv.org/abs/1603.04467.

8. Keeling MJ, Woolhouse MEJ, Shaw DJ, Matthews L, Chase-Topping M, Haydon DT, et al (2001) Dynamics of the 2001 UK foot and mouth epidemic: Stochastic dispersal in a heterogeneous landscape. Science. 294. 813–817. (doi: 10.1126/science.1065973)

9. Tildesley MJ, Deardon R, Savill NJ, Bessell PR, Brooks SP, Woolhouse MEJ et al (2008) Accuracy of models for the 2001 foot-and-mouth epidemic. Proceedings of the Royal Society of London B: Biological Sciences. 275 (1641) 1459-1468. (doi:10.1098/rspb.2008.0006)

10. Diggle, PJ. (2006) Spatio-temporal point processes, partial likelihood, foot and mouth disease.  Statistical Methods in Medical Research 2006; 15: 325-336. (doi: [10.1191/0962280206sm454oa](https://doi.org/10.1191/0962280206sm454oa))

11. Sutton, R and Barto, A (1998) Reinforcement Learning: an introduction. MIT Press.
